# Hepatoprotective effects of rice-derived peptides against acetaminophen-induced damage in mice

**DOI:** 10.3164/jcbn.16-44

**Published:** 2016-12-06

**Authors:** Kayoko Kawakami, Chie Moritani, Misugi Uraji, Akiko Fujita, Koji Kawakami, Tadashi Hatanaka, Etsuko Suzaki, Seiji Tsuboi

**Affiliations:** 1 School of Pharmacy, Shujitsu University, 1-6-1 Nishigawara, Naka-Ku, Okayama 703-8516, Japan; 2 Okayama Prefectural Technology Canter for Agriculture, Forestry and Fisheries, Research Institute for Biological Sciences (RIBS), Okayama, 7549-1 Kibichuo-cho, Kaga-gun, Okayama 716-1241, Japan; 3 SATAKE Corporation, 2-30 Saijo Nishihonmachi, Higashi-Hiroshima-shi, Hiroshima 739-8602, Japan

**Keywords:** rice-derived peptide, glutathione, acetaminophen, liver injury

## Abstract

Glutathione, the most abundant intracellular antioxidant, protects cells against reactive oxygen species induced oxidative stress and regulates intracellular redox status. We found that rice peptides increased intracellular glutathione levels in human hepatoblastoma HepG2 cells. Acetaminophen is a commonly used analgesic. However, an overdose of acetaminophen causes severe hepatotoxicity via depletion of hepatic glutathione. Here, we investigated the protective effects of rice peptides on acetaminophen-induced hepatotoxicity in mice. ICR mice were orally administered rice peptides (0, 100 or 500 mg/kg) for seven days, followed by the induction of hepatotoxicity via intraperitoneal injection of acetaminophen (700 mg/kg). Pretreatment with rice peptides significantly prevented increases in serum alanine aminotransferase, aspartate aminotransferase, and lactate dehydrogenase levels and protected against hepatic glutathione depletion. The expression of γ-glutamylcysteine synthetase, a key regulatory enzyme in the synthesis of glutathione, was decreased by treatment with acetaminophen, albeit rice peptides treatment recovered its expression compared to that achieved treatment with acetaminophen. In addition, histopathological evaluation of the livers also revealed that rice peptides prevented acetaminophen-induced centrilobular necrosis. These results suggest that rice peptides increased intracellular glutathione levels and could protect against acetaminophen-induced hepatotoxicity in mice.

## Introduction

Glutathione (γ-glutamylcysteinylglycine) is an important thiol compound with antioxidant activity that plays important roles in counteracting oxidative stress injury and maintaining cellular redox balance.^(1)^ Altered glutathione metabolism and the resulting increase in oxidative stress have been implicated in the pathogenesis of several diseases such as protein energy malnutrition, seizures, Alzheimer’s disease, Parkinson’s disease, sickle cell anaemia, chronic diseases associated with ageing and the infected state.^(2)^ Therefore, regulating glutathione biosynthesis may be a useful means of preventing such diseases. However, it is not possible to increase circulating glutathione to clinically beneficial levels through oral administration of glutathione.^(3)^

Acetaminophen (APAP) is widely used for managing pain and fever. At limited therapeutic doses, APAP is metabolized by cytochrome P450 to form the highly reactive species, *N*-acetyl-*p*-benzoquinone imine (NAPQI), which, under normal conditions, is readily detoxified by conjugation with glutathione. However, large APAP doses increase NAPQI levels, ultimately depleting glutathione levels. APAP and NAPQI may react with target proteins to form NAPQI–protein adducts, resulting in hepatic necrosis.^(4,5)^ Thus, an elevated glutathione concentration has been suggested as one of the most important strategies for treating APAP-induced hepatotoxicity. *N*-Acetylcysteine, the precursor of glutathione biosynthesis, is generally used as an antidote to APAP-induced acute liver failure in clinical practice.^(6,7)^

We have focused on the regulation of glutathione biosynthesis and have tried to discover natural compounds that increase intracellular glutathione levels. We found that rice-derived peptides increased intracellular glutathione levels. Rice is widely consumed around the world and rice-derived peptides are reported to have various biological effects such as anti-microbial activity,^(8,9)^ anti-oxidative activity^(10,11)^ and dipeptidyl peptidase-IV-inhibitory activity.^(12,13)^ Compounds that have so far been reported to increase intracellular glutathione levels include silymarin,^(14)^ cyanohydroxybutene,^(15)^ α-naphthoflavone,^(16)^ apocynin,^(17)^ epalrestat,^(18,19)^ ribose-cysteine,^(20)^ β-carotene,^(21)^ noradrenaline,^(22)^ and lactacystin.^(23)^ In a previous study, we demonstrated that rice-derived peptides (RP), obtained from commercial rice proteins using a commercial protease from *Aspergillus oryzae*, inhibit dipeptidyl peptidase-IV.^(24)^ However, there has been no follow-up report evaluating the effect of RP on intracellular glutathione levels.

In this study, we investigated the effects of RP on intracellular glutathione levels. Furthermore, we evaluated the hepatoprotective effects of RP on acetaminophen-induced hepatic damage in mice.

## Materials and Methods

### Materials

The commercial rice protein, Oryza Protein-P70, was a gift from Oryza Oil and Fat Chemical Co., Ltd. (Aichi, Japan). Denazyme AP, a protease from *Aspergillus oryzae*, was supplied from NAGASE Chemtex Co., Ltd. (Osaka, Japan). Disodium salts of bathophenanthrolinedisulfonic acid (BAPS) were purchased from Dojindo Laboratories (Kumamoto, Japan). 5,5'-Dithiobis-(2-nitrobenzoic acid) (DTNB) was bought from Wako Pure Chem. Ind., Ltd. (Osaka, Japan). NADPH and glutathione reductase (GR) from yeast were obtained from Oriental Yeast Co., Ltd. (Tokyo, Japan). All other reagents were of the highest grade available.

### Preparation of rice peptides (RP)

RP was prepared as previously described.^(24)^ Two grams of rice protein was dispersed in 40 ml of distilled water, adjusted to pH 7.5 with 5 M NaOH, and incubated with 1% (w/w) Denazyme AP at 50°C for 17 h. The resulting hydrolysates were heated at 80°C for 30 min to inactivate the proteases. The hydrolysates were subsequently centrifuged at 2,000 × *g* for 20 min. The supernatants were freeze-dried and stored at 4°C for further studies.

### Cell culture

The human hepatoblastoma cell line HepG2 was obtained from RIKEN BRC Cell Bank (Ibaraki, Japan). The cells were cultured in a Minimum Essential Medium (MEM) (Sigma-Aldrich, St. Louis, MO) containing 10% fetal bovine serum, 25 mM HEPES (pH 7.4), 0.56 µg/ml amphotericin B, 100 U/ml penicillin, and 100 µg/ml streptomycin at 37°C in a humidified atmosphere of 5% CO_2_. The cells were seeded at 3 × 10^5^ cells/well in a 6-well plate (Thermo Fisher Scientific, Waltham, MA).

### Measurement of intracellular glutathione

HepG2 cells were cultured for 48 h and treated with RP (0, 2.5, 5 and 10 mg/ml) for 24 h. After the treatment, the cells were collected and homogenized in 0.1 M HCl containing 1 mM BAPS. After deproteinization, the obtained supernatants were used for measuring the total glutathione (reduced and oxidized form) content. Intracellular glutathione levels were measured using Matsumoto’s method^(25)^ and protein concentrations were determined using Bradford’s protein assay kit (Bio-Rad Laboratories, Hercules, CA). In brief, 80 µl of the sample solution was mixed the reaction mixtures included 40 µl of 1.05 mM NADPH, 20 µl of 6 mM DTNB and 0.04 µl of 100 U/L of GR, in 125 mM sodium phosphate (pH 7.5) and 6.3 mM EDTA. The change in the optical density was followed at 405 nm. Each glutathione concentration value was normalized to the protein concentration of sample.

### Animals

Three-week-old male ICR mice were purchased from Japan SLC (Shizuoka, Japan). The animals were housed at 24 ± 1°C under a 12 h light/dark cycle and had free access to a standard diet (MF, Oriental Yeast Co., Ltd.) and distilled water for one week prior to the experiment. Animal experimental protocols were approved by the Animal Experimentation Committee of Shujitsu University and the study was conducted in accordance with the Animal Experimentation Guidelines of Shujitsu University.

The mice were randomly assigned to four groups. The RP-treated group was orally administered RP (100 and 500 mg/kg body weight) daily using a stomach tube for seven days. Saline was administered to the control and APAP groups. All groups fasted for 16 h before being injected intraperitoneally with APAP (700 mg/kg).

### Biochemical analysis

At 4 and 6 h after APAP treatment, the mice were sacrificed and their blood samples were collected and centrifuged at 750 × *g* for 10 min at 4°C. Serum alanine aminotransferase (ALT) and aspartate aminotransferase (AST) levels were measured using a Transaminase CII Test Wako kit (Wako Pure Chem.). The result is shown in Karmen unit. Serum lactate dehydrogenase (LDH) was estimated using a Cytotoxicity Detection Kit^PLUS^ (LDH) obtained from Roche Applied Science (Mannheim, Germany).

Livers from sacrificed mice were immediately washed in 0.9% NaCl solution and homogenized in ice-cold 0.1 M HCl containing 1 mM BAPS. After deproteinization, the total glutathione contents were measured as described above.

### Histopathologic examination

After 6 h of APAP treatment, the liver was fixed in 10% phosphate-buffered neutral formalin, dehydrated in graded (50–100%) alcohol, and embedded in paraffin. Thin sections were cut and stained with hematoxylin and eosin (H&E) stain for photomicroscopic assessment.

### Western blotting analysis

For the preparation of protein extracts, HepG2 cells or livers were homogenized in RIPA buffer (Santa Cruz Biotechnology, Santa Cruz, CA). After incubation for 30 min at 4°C, the homogenates were centrifuged at 18,800 × *g* for 10 min at 4°C. Protein concentrations in the tissue homogenates were determined using the Bio-Rad protein assay. For immunoblot analysis, the prepared proteins were separated by SDS-PAGE and transferred electrophoretically to a PVDF membrane. The membrane was blocked by a PVDF Blocking Reagent (Toyobo, Osaka, Japan) for 1 h at room temperature. Immunoblotting was performed using primary antibodies against the γ-GCS heavy subunit (γ-GCSh), the γ-GCS light subunit (γ-GCSl), cytochrome P450 2E1 (CYP2E1) (Santa Cruz Biotechnology) and β-actin (Sigma-Aldrich) for 1 h at room temperature. Bound antibodies were detected using a secondary peroxidase-conjugated anti-rabbit or anti-mouse IgG (GE Healthcare, Buckinghamshire, England). Target proteins were visualized using an ECL reaction solution (GE Healthcare).

### Statistics

The data were expressed as the mean ± SEM. Statistical analysis of the data was performed with ANOVA, followed by Student’s *t* test to identify differences among groups. Differences were considered significant at *p*<0.05.

## Results

### Effect of RP treatment on intracellular glutathione and γ-GCS levels

**Fig. 1:**
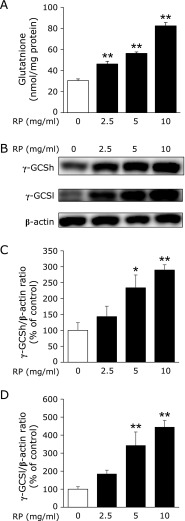
The effect of RP on total glutathione levels and γ-GCS expression in HepG2. Cells were incubated with the indicated concentrations of RP for 24 h. (A) Total glutathione levels. The values given are the mean ± SEM (*n* = 6). **p<0.01 vs RP-untreated control. (B) Western blot analysis of γ-GCS protein levels. (C, D) Percentage of target protein/β-actin was calculated by comparing to RP untreated control. The values given are the mean ± SEM (*n* = 4). **p<0.01 and *p<0.05 vs RP-untreated control.

RP treatment increased intracellular glutathione levels in a dose-dependent manner (Fig. 1A). The intracellular glutathione level, after treatment with 10 mg/ml RP, was 82.4 ± 7.6 nmol/mg protein, which was 2.7-fold higher than the control that had a mean of 30.4 ± 3.2 nmol/mg protein. We further investigated the expression of γ-GCSh and γ-GCSl proteins at 24 h after RP treatment. RP treatment induced the expression of γ-GCSh and γ-GCSl proteins in a dose-dependent manner (Fig. 1B). Expression levels of γ-GCSh and γ-GCSl proteins were enhanced to 2.9- and 4.5-fold, respectively, by 10 mg/ml RP treatment (Fig. 1C and D).

### Effect of RP on the biomarkers of hepatocellular damage

**Fig. 2:**
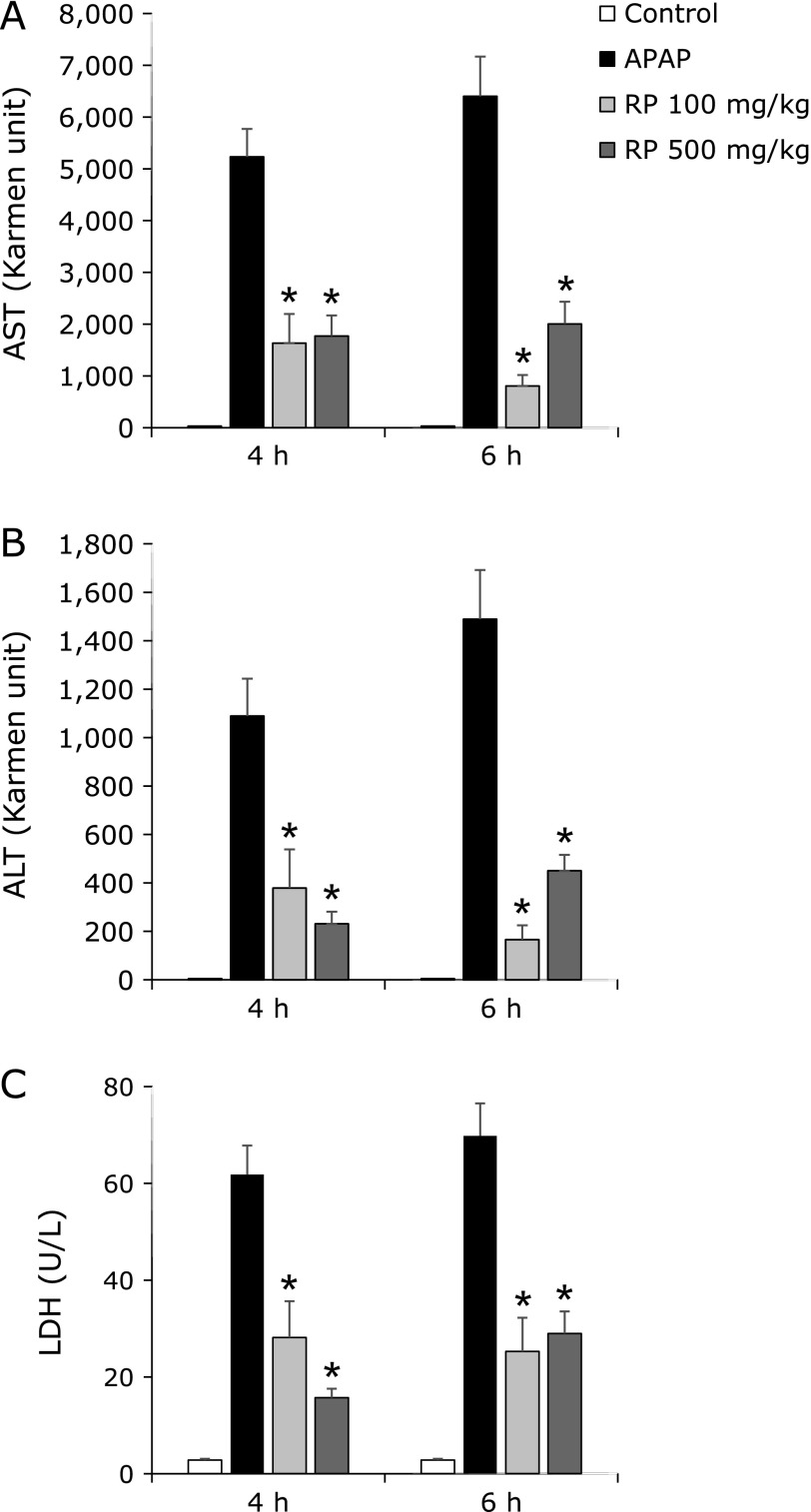
The effect of RP on serum AST (A), ALT (B) and LDH (C) in mice with an acetaminophen-induced liver injury. The values given are the mean ± SEM (*n* = 5–6). *p<0.01 vs APAP treated group.

We afterward assessed the protective effects of RP on APAP-induced serum AST, ALT and LDH levels. A marked change in body weight was not observed between the groups for seven days (data not shown). Treatment with APAP at 4 h markedly increased serum AST (Fig. 2A), ALT (Fig. 2B) and LDH (Fig. 2C) levels, which reached 5,229.5 ± 540.2 Karmen unit, 1089.1 ± 154.3 Karmen unit and 61.8 ± 6.0 U/L respectively while the control group had levels of 30.8 ± 3.0 Karmen unit, 4.5 ± 0.4 Karmen unit and 2.8 ± 0.3 U/L respectively. The administration of RP alone did not affect serum AST, ALT and LDH levels (data not shown). However, pretreatment with RP significantly inhibited the elevation of serum AST, ALT, and LDH levels. At 6 h after APAP treatment, RP also significantly inhibited the elevation of these serum enzymes.

### Effect of RP treatment on hepatic glutathione levels

**Fig. 3:**
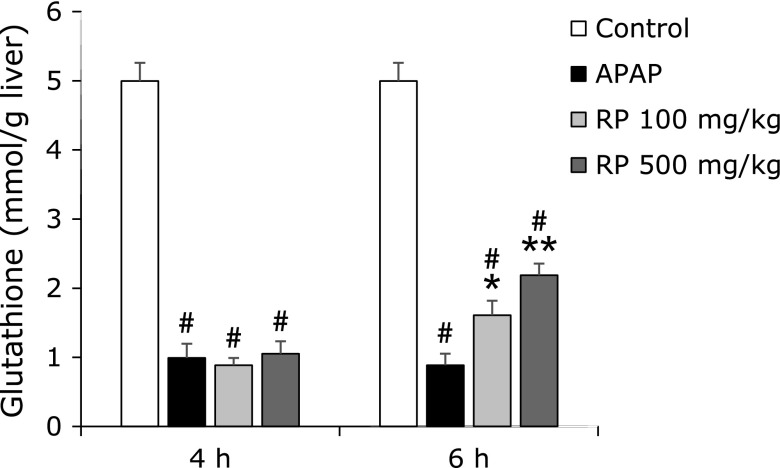
The effect of RP on hepatic glutathione levels in mice with an acetaminophen-induced liver injury. The values given are the mean ± SEM (*n* = 5–6). ^#^*p*<0.01 vs control group. **p<0.01 and *p<0.05 vs APAP treated group.

The APAP-treated group had significantly decreased glutathione concentrations than that of the control group (Fig. 3). At 4 h after APAP treatment, a marked change was not observed between the groups. However, pretreatment with RP dose-dependently recovered the APAP-induced glutathione depletion at 6 h after APAP treatment.

### Histopathological examination of liver sections

**Fig. 4:**
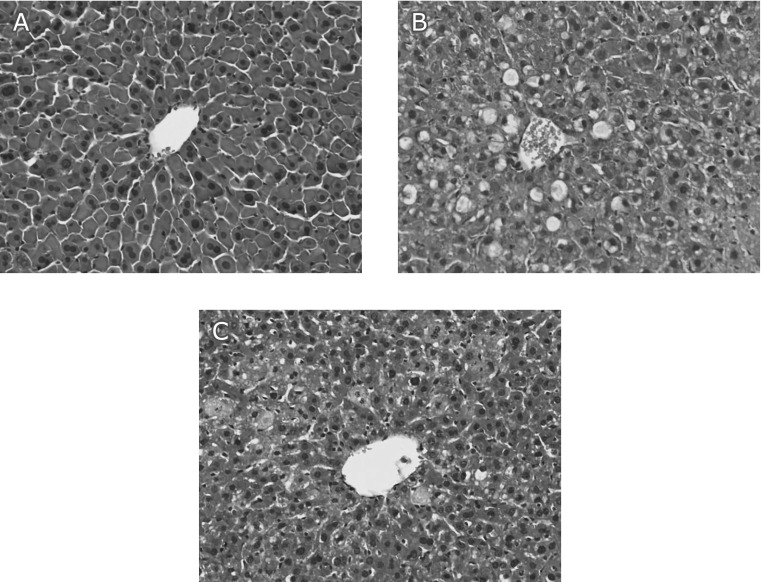
H&E staining of the livers after acetaminophen treatment. Typical images were chosen from the different experimental groups (original magnification ×20). (A) Control group: normal lobular architecture and cell structure, (B) APAP group: multiple and extensive areas of portal inflammation and hepatocellular necrosis and a moderate increase in inflammatory cells’ infiltration, (C) RP (100 mg/kg)-treated acetaminophen group: minimal hepatocellular necrosis and inflammatory cells’ infiltration and mild portal inflammation.

 The histological features shown in Fig. 4 illustrate the lobular architecture and cell structure of normal livers from the control animals. However, the APAP-exposed liver showed multiple and extensive areas of portal inflammation, hepatocellular necrosis that was randomly distributed throughout the parenchyma and a moderate increase in inflammatory cells’ infiltration. These pathological changes were ameliorated by the RP treatment.

### Effect of RP treatment on γ-GCS and CYP2E1 protein expression in mice liver

**Fig. 5:**
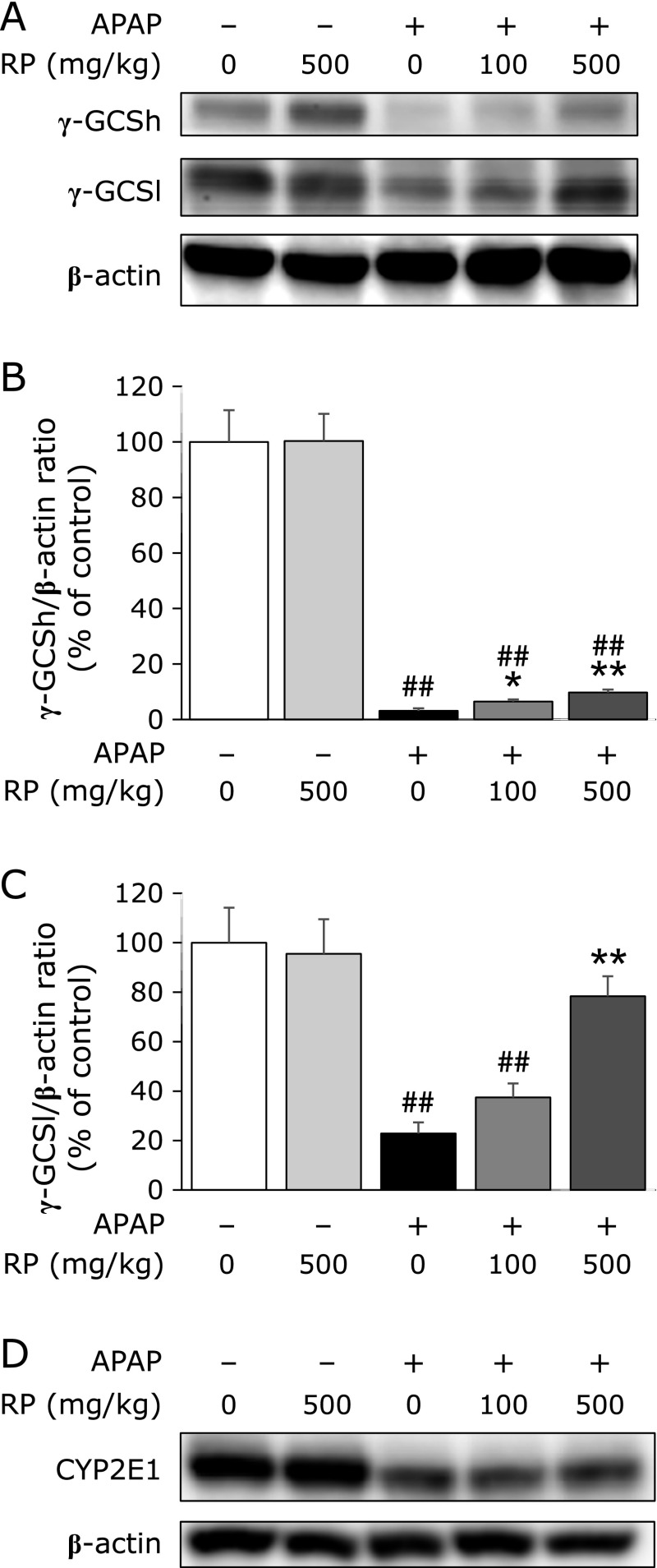
The expression level of γ-GCS and CYP2E1 in mice liver. Protein extracts from liver tissue were analyzed by SDS-PAGE and immunoblotting by using antibodies against γ-GCSh, γ-GCSl and CYP2E1, respectively. (A) Western blot analysis of γ-GCS protein levels at 6 h after APAP treatment. Lane 1, Control; lane 2, RP 500 mg/kg; lane 3, APAP; lane 4, APAP + RP 100 mg/kg; and lane 5, APAP + RP 500 mg/kg. (B, C) Percentage of target protein/β-actin was calculated by comparing to RP untreated control. The values given are the mean ± SEM (*n* = 5–6). ^##^*p*<0.01 vs control group. **p<0.01 and *p<0.05 vs APAP treated group. (D) Western blot analysis of CYP2E1 protein levels at 6 h after APAP treatment.

We further observed the expression of γ-GCSh and γ-GCSl proteins at 6 h after APAP treatment. The APAP-treated group had significantly decreased expression of γ-GCSh and γ-GCSl proteins than the control group (Fig. 5A, lane 3). On the other hand, RP dose-dependently recovered γ-GCSh and γ-GCSl protein expression (Fig. 5B and C). No significant difference in CYP2E1 protein expression was observed between the APAP-treated group and APAP + RP treated group (Fig. 5D).

## Discussion

Altered glutathione metabolism, in association with increased oxidative stress, has previously been implicated in the pathogenesis of many diseases.^(2)^ Therefore, regulating glutathione biosynthesis may be a useful approach in preventing such diseases. In the present study, RP increased intracellular glutathione levels. Here, we investigated the protective effects of RP against APAP-induced hepatotoxicity in mice.

We demonstrated that intracellular glutathione was significantly increased by RP treatment (Fig. 1A). Glutathione is a tripeptide synthesized from glutamate, cysteine and glycine. We reported that RP concentrations of glutamate, cysteine and glycine were 24.1, 3.0 and 28.3 nmol/mg of RP, respectively.^(24)^ However, RP does not particularly include more of these amino acids than that of other foods. In our previous study, the molecular weights of RP were estimated to be around 151 Da, which corresponds to the weight of a di- or a tripeptide.^(24)^ These results suggested that RP, unlike glutathione precursors, might act through a more efficient and effective mechanism to elevate intracellular glutathione levels. Several studies have reported that intracellular glutathione levels were increased by food-derived products such as proteins,^(26)^ polyphenols^(27,28)^ and carotenoids.^(21)^ This study is the first to report that rice-derived peptides increase intracellular glutathione levels.

We assessed the effect of oral administration of RP on APAP-induced liver injury. The levels of serum AST, ALT and LDH are the most sensitive biomarkers that can directly indicate the extent of hepatic damage.^(29)^ APAP caused a significant increase in the levels of AST, ALT and LDH (Fig. 2). However, pretreatment with RP significantly decreased the serum levels of these enzymes. Moreover, results from histological observations of the H&E stain also showed that RP decreased hepatocyte necrosis significantly (Fig. 4).

APAP evokes hepatotoxicity after its biotransformation to a reactive metabolite, known as NAPQI. APAP is metabolized through an enzymatic reaction mediated by the cytochrome P450 system, engendering NAPQI.^(30)^ Especially CYP2E1 is the main metabolic enzyme for converting APAP into NAPQI.^(31)^ To investigate whether the protective effect of RP on the liver are associated with the inhibition of CYP2E1, we measured the expression of CYP2E1 protein expression. The result showed that RP did not inhibit CYP2E1 protein expression (Fig. 5D). Our data indicated CYP2E1 is not involved in the hepatoprotective effect of RP. NAPQI, a toxic metabolite of APAP, is detoxified by glutathione to form APAP-glutathione adducts.^(32)^ Due to the important role played by glutathione in the antioxidant defence system, it could become a key determinant of APAP-induced hepatotoxicity. Therefore, we measured hepatic glutathione levels. In the present study, the APAP group had significantly decreased hepatic glutathione levels compared to the control group (Fig. 3). Pretreatment with RP dose-dependently increased glutathione levels at 6 h after APAP treatment. However, a marked change was not observed between the groups at 4 h after APAP treatment. In addition, administration of RP alone did not affect hepatic glutathione levels compared to that of the control (data not shown). Glutathione is an abundant cellular antioxidant and is maintained at a certain level in cells.^(33)^ These results suggest that RP may accelerate hepatic glutathione recovery.

Glutathione synthesis is controlled by two rate-limiting factors. One is the γ-GCS enzyme, which catalyzes the first reaction step in glutathione synthesis. γ-GCS consists of a heavy catalytic subunit (γ-GCSh) and a light regulatory subunit (γ-GCSl), which are encoded by different genes.^(34)^ Expression of γ-GCS proteins was observed at 6 h after APAP treatment, similarly, RP recovered hepatic glutathione levels at 6 h after APAP treatment. A previous study reported that γ-GCS protein expression decreased after APAP treatment,^(35)^ which was also observed in this study where APAP decreased the expression of γ-GCS proteins (Fig. 5A). However, RP treatment dose-dependently recovered the expression of γ-GCS proteins than the APAP treatment. Induction of γ-GCS is mainly due to a transcriptional activation mediated by the nuclear factor, erythroid 2-related factor 2 (Nrf2).^(36)^ Additionally, the Nrf2-dependent antioxidant defence system composed of glutathione, phase II detoxifying enzymes and reactive oxygen species inactivating-enzymes play a key role in protecting cells from oxidative damage by NAPQI. Moreover, a previous study reported that Nrf2-mediated gene regulation is efficacious in protecting hepatocytes against APAP-induced hepatotoxicity.^(37)^ Further studies are required to evaluate the effects of RP on the Nrf2 pathway.

We demonstrated that RP significantly increased intracellular glutathione. Moreover, our results suggested that RP has a potent hepatoprotective effect on APAP-induced liver injury in mice. It is expected that natural products would have a lower risk of side effects than chemical agents. RP are hydrolysates derived from rice, which is a staple food in many countries worldwide. Thus, administration of rice peptides would present lower risks of side effects than the administration of other chemical agents. Our findings indicate that RP could possibly play important roles in preventing oxidative stress-associated diseases by regulating glutathione biosynthesis.
